# Peripartum myocardial infarction associated with coronary spasm and acquired protein S deficiency: A case report

**DOI:** 10.1097/MD.0000000000018108

**Published:** 2019-11-27

**Authors:** Yoshiaki Oshima, Kazumasa Yamasaki, Akihiro Otsuki, Masato Nakasone, Ryo Endo, Naoki Moriyama, Seiji Sakamoto, Yukari Minami, Yoshimi Inagaki

**Affiliations:** a Department of Anesthesiology, Yonago Medical Center; b Division of Anesthesiology and Critical Care Medicine, Department of Surgery, Tottori University Faculty of Medicine, Tottori, Japan.

**Keywords:** acquired protein S deficiency, acute myocardial infarction, coronary spasm, peripartum period, pregnancy

## Abstract

**Rationale:**

Coronary angiography (CAG) findings of acute myocardial infarction (AMI) in pregnant women are characterized by a high incidence of normal coronary arteries. This is the first report of AMI with normal coronary arteries during pregnancy, showing coronary spasm and pregnancy-related acquired protein S (PS) deficiency.

**Patient concerns:**

A 30-year-old Japanese woman was admitted to an emergency department. One hour before admission, she developed sudden onset of precordial discomfort, back pain, and dyspnea. She was a primigravida at 39 weeks’ gestation and had no abnormality in the pregnancy thus far. She had no history of heart disease, diabetes, hypertension, dyslipidemia, deep vein thrombosis (DVT), smoking, or oral contraceptive use and no family history of ischemic heart disease, hemostasis disorder, or DVT. She did not take any medication.

**Diagnosis:**

Electrocardiography showed ST-segment elevations in leads II, III, aVF, and V2-V6. Heart-type fatty acid-binding protein was positive. Echocardiography showed hypokinesis of the anterior interventricular septum and inferior wall. Continuous intravenous infusion of isosorbide dinitrate was initiated. Coronary computed tomography angiography revealed diffuse narrowing of the apical segment of the left anterior descending coronary artery. Three hours after admission, troponin T became positive, and the following enzymes reached their peak levels: creatine kinase (CK), 1,886 U/L; CK-muscle/brain, 130 U/L. She was diagnosed with transmural AMI due to severe coronary spasm and administered benidipine hydrochloride. Five hours after admission, premature membrane rupture occurred.

**Interventions:**

Emergency cesarean section was performed. There were no anesthetic or obstetrical complications during the operation. On postpartum day 1, the free PS antigen level was low (29%). On postpartum day 18, she was discharged with no reduction in physical performance.

**Outcomes:**

Four months after the infarction, CAG showed normal coronary arteries. Acetylcholine provocation test showed diffuse vasospasm in the coronary artery. She was advised that her next pregnancy should be carefully planned. Two years after delivery, free PS antigen level was within normal range, at 86%. She had not experienced recurrence of angina during the 2-year period. Her child was also developing normally.

**Lessons:**

In addition to coronary spasm, pregnancy-related acquired PS deficiency may be involved in AMI etiology.

## 1 Introduction

Acute myocardial infarction (AMI) occurs in 2.8 to 6.2 women per 100,000 deliveries.^[1,2]^ Traditional coronary risk factors were absent in 43% of patients with AMI during pregnancy.^[3]^ Coronary lesions involved in AMI during pregnancy consisted of coronary dissection (43%), arteriosclerosis (27%), arteriosclerosis-free thrombosis (17%), and normal blood vessels (11%).^[4]^ Coronary angiography (CAG) findings of AMI in pregnant women are characterized by a low incidence of arteriosclerosis, which is a common cause of AMI, and a high incidence of coronary dissection and normal coronary arteries.^[4]^ To our knowledge, this is the first report of AMI with normal coronary arteries during pregnancy, showing coronary spasm and pregnancy-related acquired protein S (PS) deficiency.

## 2 Case report

A 30-year-old Japanese woman (152 cm, 59 kg) was admitted to our emergency department. One hour before admission, she developed sudden onset of precordial discomfort, back pain, and dyspnea. She was a primigravida at 39 weeks’ gestation and had no abnormality in the pregnancy progressed thus far. She had no history of heart disease, diabetes, hypertension, dyslipidemia, coagulation/hemostasis disorder, deep vein thrombosis (DVT), smoking, substance abuse, or oral contraceptive use and no family history of ischemic heart disease, hemostasis disorder, or DVT. She did not take any medication.

On physical examination, her blood pressure was 168/96 mm Hg, pulse rate was 64 bpm, heart and breath sounds were normal, and no peripheral edema was noted. Electrocardiography (ECG) showed ST-segment elevations in leads II, III, aVF, and V2-V6. The chest plain radiograph was normal, and laboratory results were as follows: platelet count, 186 × 10^9^/L; hemoglobin, 12.1 g/dL; white blood cell count, 10400/μL; C-reactive protein, 0.32 mg/dL (normal range <0.20); heart-type fatty acid-binding protein, positive; troponin T, negative; creatine kinase (CK), 26 U/L; CK-muscle/brain (MB), 4 U/L; aspartate transaminase, 17 U/L; and lactic dehydrogenase, 200 U/L. Echocardiography revealed severe hypokinesis in the mid anteroseptal segment and the apical anteroseptal and inferior segments, and the left ventricular ejection fraction (LVEF) was reduced to 40%. Continuous intravenous infusion of isosorbide dinitrate was initiated. ECG showed reduction of the ST-segment elevation, and echocardiography showed improvement of the left ventricular wall motion abnormalities.

We performed triple-rule-out coronary computed tomography (CT) angiography (CCTA), which revealed no aortic dissection or pulmonary emboli, although diffuse narrowing of the apical segment of the left anterior descending coronary artery was present. Triple rule-out CCTA provides noninvasive visualization of coronary arteries with simultaneous evaluation of the pulmonary arteries and thoracic aorta. Three hours after admission, troponin T became positive, and the levels of the following enzymes reached their peaks: CK, 1886 U/L; CK-MB, 130 U/L; aspartate transaminase, 140 U/L, and lactic dehydrogenase, 414 U/L. She was diagnosed with transmural AMI due to severe coronary spasm and administered benidipine hydrochloride 8 mg/d.

Five hours after admission, the premature rupture of membranes occurred. Seven hours after admission, she was transferred to the operating room for emergency cesarean section. We monitored her with ECG, pulse oximetry, and invasive arterial pressure. First, an epidural catheter was inserted through the T9-10 interspace. Next, a 25G spinal needle was inserted through the L5-S interspace, and 8 mg of hyperbaric bupivacaine was injected. Since sensory block was achieved only at T10, 5 mL of 1% lidocaine was injected via the epidural catheter. Cesarean section was started when the sensory block reached T5. A 2930 g baby was born with Apgar scores of 8 and 9 at 1 and 5 minutes, respectively. Oxytocin 5 U was injected into the myometrium. There were no anesthetic or obstetrical complications during the operation.

At postpartum day 1, thrombophilia screening yielded negative results for antinuclear antibody, anticardiolipin immunoglobulin G, and lupus anticoagulant. Furthermore, protein C activity was within the normal range (76% [normal range 64–146]), the level of free PS antigen was low (29% [normal range 60–150]), and the level of thrombin anti-thrombin III complex was high (4.1 ng/mL [normal range <3.0]).

At postpartum day 13, echocardiography revealed that while the LVEF was 54%, there was a left ventricular thrombus (LVT) adjacent to the area of apical hypokinesis. The platelet count measured on the same day was high (631 × 10^9^/L), and we continuously infused heparin and administered warfarin. At postpartum day 18, she was discharged with no reduction in physical performance. At postpartum day 49, follow-up echocardiography demonstrated disappearance of the LVT, and the platelet count was 292 × 10^9^/L.

Four months after infarction, we performed CAG, which showed normal coronary arteries. The provocation test with acetylcholine (Ach) showed 90% diffuse vasospasm in the right coronary artery. Ach was not injected into the left coronary artery considering the risk of ventricular fibrillation.

**Figure 1:**
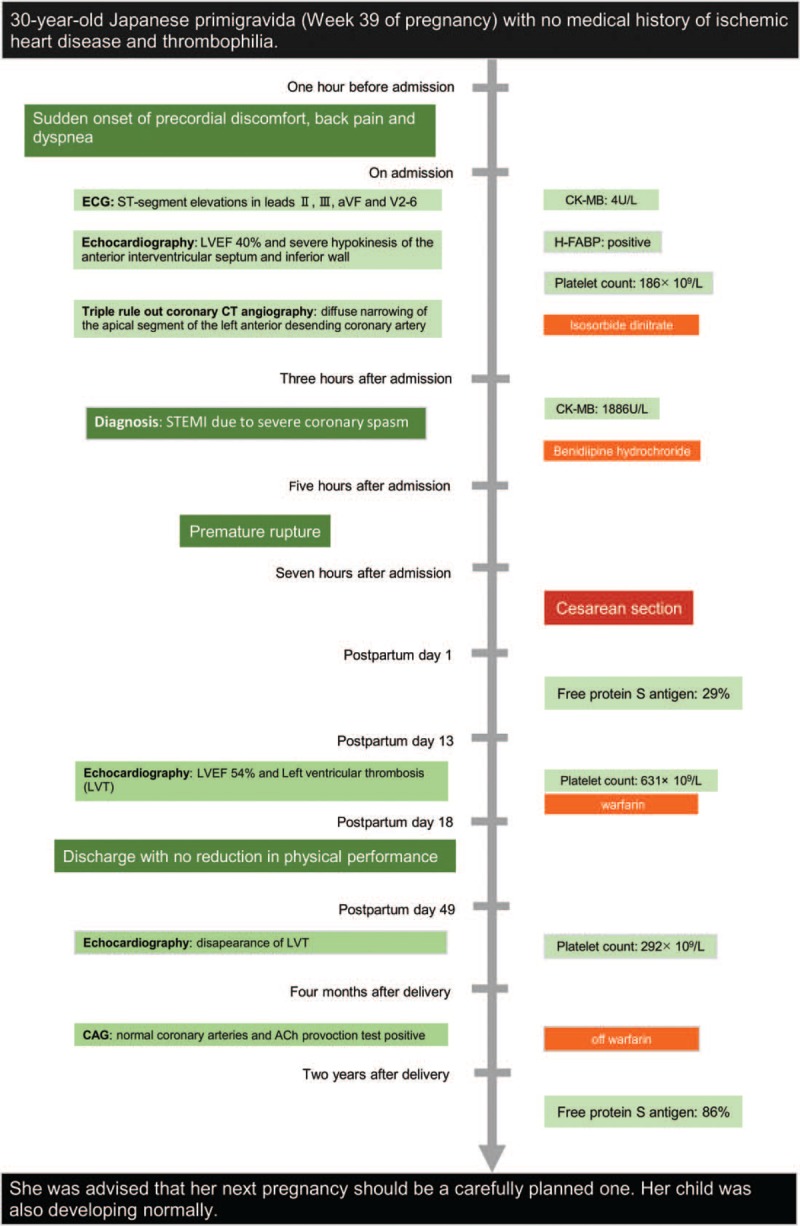
Timeline for the case.

We advised her that her next pregnancy should be a carefully planned one. Two years after delivery, the level of free PS antigen was within the normal range, at 86%. She had not experienced recurrence of angina during the 2-year period. Her child was also developing normally. The timeline for this case is summarized in Figure 1.

## 3 Discussion

Myocardial infarction with normal coronary arteries (MINCA) can be classified as with and without risk factors for ischemic heart disease. Etiological factors for MINCA may include prolonged coronary spasm, coronary thrombosis with spontaneous lysis, cocaine abuse, viral myocarditis, aortic dissection, hypercoagulable states, autoimmune vasculitis, and carbon monoxide poisoning.^[5]^ At the time of coronary spasm, enhancement of the coagulation system may occur in the coronary circulation.^[6]^ Some cases of coronary spasm-induced thrombosis leading to AMI have been reported.^[7–13]^ Furthermore, prolonged coronary spasm during a spasm provocation test resulted in intracoronary thrombus formation, leading to AMI, in 1 case.^[14]^ Etsuda et al^[15]^ demonstrated intracoronary thrombus formation at the time of coronary spasm using coronary angioscopy in 1 of 10 patients.

PS is a coenzyme for protein C. Activated protein C (APC) inactivates activated factors V and VIII in the presence of PS, exhibiting anticoagulant actions. The resistance to the anticoagulant actions is termed “APC resistance,” which results in thrombophilia. In Caucasian populations, the factor V Leiden mutation is primarily involved in the etiology of congenital APC resistance.^[16]^ In Japan, the PS Tokushima mutation is primarily involved.^[17]^ Patients with hetero-type congenital PS deficiency primarily develop DVT after adolescence.^[18]^ Moreover, in those aged ≤55 years, the risk of arterial thrombosis increases 5.7-fold.^[19]^ In Caucasian populations, the factor V Leiden mutation was frequently detected in 55-year-old or younger patients diagnosed with MINCA.^[20]^

Coronary spasm or PS deficiency involved in the onset of AMI was also reported in a 17-year-old girl.^[21]^ She was treated successfully with thrombolytic therapy. CAG after symptom relief showed normal coronary arteries. The free PS level at the time of AMI onset was 40%. After 2 months, it was 60%, being within the normal range. The free PS levels among members of her family were normal, and she had not been diagnosed with congenital PS deficiency. However, the cause of the temporary decrease in her free PS level was unknown. Our case did not undergo thrombolytic therapy because thrombolytic therapy increases the incidence of fetal and maternal hemorrhage.

Pregnancy is an acquired factor for APC resistance. A study revealed that PS activity reduced during the course of pregnancy, reaching a minimum at the time of delivery.^[22]^ Furthermore, the mean free PS level during the second and third trimesters was reported to be 38%, being below the lower limit of the normal range.^[23]^ PS activity was observed to normalize (up to 91%) 6 weeks after delivery.^[24]^ Therefore, a diagnosis of congenital PS deficiency should be made ≥6 weeks after delivery.^[24]^

In the present case, although the patient's free PS antigen level was below the normal lower limit during pregnancy, in the second year after delivery, her free PS antigen level was normal; therefore, a diagnosis of acquired, rather than congenital, PS deficiency was made. Many young patients with MINCA have hypercoagulable states. However, all patients with MINCA do not have coagulation abnormalities. It appears that the hypercoagulable states in itself are not sufficient to cause MINCA. The final reasonable cause for MINCA, which seems to have sufficient evidence in support of its inclusion as a factor, is coronary spasm. However, we could not find a case-control study that investigated whether acquired PS deficiency causes myocardial infarction (MI). Furthermore, few studies have examined whether PS deficiency causes MI without differentially diagnosing if the cause of PS deficiency is congenital or acquired. The sample size of these studies was small.^[19,25–30]^ In a case-control study that compared MINCA with MI with obstructive coronary artery disease (MICAD), the incidence of PS deficiency was 4% in patients with MINCA and 0% in patients with MICAD, without a statistically significant difference between these patient groups (*P* = .47). Furthermore, this study's major limitation was a very small sample size (25 patients).^[25]^ In another case-control study that compared 50 patients with MI aged <55 years with a healthy control group, the incidence of PS deficiency was 24% in patients with MI and 0% in the healthy control group, with a statistically significant difference between these patient groups (*P* = .001).^[26]^ A recent review concluded that the risk of MI was not negligible in patients with PS deficiency.^[31]^ Coronary spasm-induced intracoronary thrombosis may have been enhanced by the presence of acquired PS deficiency, possibly causing AMI during her pregnancy.

LVT after AMI is primarily related to poor LVEF and regional asynergy. It frequently occurs at the site of wall motion abnormalities of the apex.^[32]^ The incidence of LVT was reported as 32% in patients with a LVEF of ≤30% and 3% in those with a LVEF of 50% to 59%.^[33]^ The possible involvement of the factor V Leiden mutation in the onset of LVT after AMI was examined, but its involvement was not confirmed.^[34]^ A case of essential thrombocythemia leading to AMI and subsequent LVT was also reported.^[35]^ In the present case, LVT occurred despite preserved LVEF, suggesting the involvement of secondary thrombocytosis after cesarean section as an etiological factor.

Appropriate use criteria guidelines for cardiac CT^[36]^ recommend that CCTA should be indicated for low- to middle-risk patients in whom acute coronary syndrome (ACS) is suspected despite the lack of an ST-segment elevation on ECG or a significant increase in the myocardial enzyme level. Although our patient was a high-risk patient in whom ACS was suspected, we thought she did not have ACS with obstructive atherosclerosis, because she had few risk factors for MI before onset. Accordingly, we initially performed triple rule-out CCTA to rule out pulmonary embolism or aortic dissociation. Elkayam et al^[4]^ emphasized that CAG should be carefully performed in pregnant women, and that indications for percutaneous coronary intervention should be limited to occlusion of the proximal segment. In 5 of 69 pregnant women with AMI, the infusion of contrast medium for CAG led to iatrogenic coronary dissection.^[4]^ The 5 patients underwent coronary artery bypass grafting, but 1 died.^[4]^

With respect to regional anesthesia for cesarean section in the presence of MI or peripartum cardiomyopathy, spinal anesthesia by a single-shot injection is contraindicated,^[37]^ and analgesia level titration by epidural anesthesia,^[37]^ continuous spinal anesthesia,^[37]^ or combined spinal-epidural anesthesia^[38]^ is recommended.

## 4 Conclusion

We encountered a primigravida (week 39 of pregnancy) with AMI in whom coronary spasm, as well as pregnancy-related, acquired PS deficiency may have been involved in the etiology of AMI. This is the first report of MINCA during pregnancy, showing coronary spasm and pregnancy-related acquired PS deficiency.

## Acknowledgment

The authors thank Editage (www.editage.jp) for English language editing.

## Author contributions

**Conceptualization:** Yoshiaki Oshima, Kazumasa Yamasaki.

**Data curation:** Masato Nakasone.

**Investigation:** Ryo Endo.

**Project administration:** Seiji Sakamoto.

**Supervision:** Yukari Minami.

**Visualization:** Naoki Moriyama.

**Writing – original draft:** Yoshiaki Oshima.

**Writing – review and editing:** Akihiro Otsuki, Yoshimi Inagaki.
